# Socio-demographic determinants and effect of structured personal diabetes care: a 19-year follow-up of the randomized controlled study diabetes Care in General Practice (DCGP)

**DOI:** 10.1186/s12902-017-0227-x

**Published:** 2017-12-08

**Authors:** Andreas Heltberg, Volkert Siersma, John Sahl Andersen, Christina Ellervik, Henrik Brønnum-Hansen, Jakob Kragstrup, Niels de Fine Olivarius

**Affiliations:** 1 Section of General Practice, Institute of Public Health, Faculty of Health Sciences, University of Copenhagen, Øster Farimagsgade 5, Copenhagen, Denmark; 2 The Research Unit for General Practice, Department of Public Health, University of Copenhagen, Copenhagen, Denmark; 3 Department of Production, Research, and Innovation, Sorø, Region Zealand Denmark; 4 Department of Laboratory Medicine, Boston Children’s Hospital Study, Boston, MA USA; 5 Department of Clinical Medicine, Faculty of Health and Medical Sciences, University of Copenhagen, Copenhagen, Denmark; 6 Department of Social Medicine, University of Copenhagen, Copenhagen, Denmark

**Keywords:** Type 2 diabetes mellitus, Social inequalities, Intervention, Clinical outcomes

## Abstract

**Background:**

We investigated how four aspects of socio-demography influence the effectiveness of an intervention with structured personal diabetes care on long-term outcomes.

**Methods:**

The Diabetes Care in General Practice (DCGP) study is a cluster-randomized trial involving a population-based sample of 1381 patients with newly diagnosed type 2-diabetes mellitus. We investigated how education, employment, cohabitation status and residence influenced the effectiveness of 6 years of intervention with structured personal diabetes care, resembling present day recommendations. Outcomes were incidence of any diabetes-related endpoint and death during 19 years after diagnosis, and cardiovascular risk factors, behaviour, attitudes and process-of-care variables 6 years after diagnosis.

**Results:**

Structured personal care reduced the risk of any diabetes-related endpoint and the effect of the intervention was modified by geographical area (interaction *p* = 0.034) with HR of 0.71 (95%CI: 0.60–0.85) and of 1.07 (95%CI: 0.77–1.48), for patients in urban and rural areas, respectively. Otherwise, there was no effect modification of education, employment and civil status on the intervention for the final endpoints. There were no noticeable socio-demographic differences in the effect of the intervention on cardiovascular risk factors, behaviour, attitudes, and process-of-care.

**Conclusion:**

Structured personal care reduced the aggregate outcome of any diabetes-related endpoint and independent of socio-demographic factors similar effect on cardiovascular risk factors, behaviour, attitudes and process of care, but the intervention did not change the existing inequity in mortality and morbidity. Residence modified the uptake of the intervention with patients living in urban areas having more to gain of the intervention than rural patients, further investigations is warranted.

**Trial registration:**

ClinicalTrials.gov registration no. NCT01074762 (February 24, 2010).

**Electronic supplementary material:**

The online version of this article (10.1186/s12902-017-0227-x) contains supplementary material, which is available to authorized users.

## Background

Epidemiological studies have repeatedly shown increased occurrence of type 2 diabetes mellitus (T2DM) among people with low socioeconomic status [1, 2] and living in rural areas [3]. Primarily due to improved diabetes care, mortality of T2DM patients has decreased substantially in recent decades [4], although this trend has been less favourable among those with low socio-economic status (SES) [5–10]. A recent Scandinavian study showed increased mortality among people of low SES, but could not show any systematic differences in mortality between patients living in rural and urban areas [8].

Social inequality in mortality and morbidity of patients with T2DM can only be partially explained by differences in the increased incidence of other comorbid disease [1, 11]. SES has been reported to influence metabolic control [12], pharmacological treatment [13] and ability to change lifestyle according to the recommendations [14, 15]. Inequality in access to and utilization of health care [16, 17] could influence the course of T2DM [18, 19].

Though SES and place of living influence care and prognosis of T2DM [8, 10, 11, 20], there is only limited evidence as to whether the effectiveness of diabetes interventions differs with regard to SES and residence. Recent reports suggest that interventions with intensive diabetes care diminish the socio-economic differences in intermediate outcomes [13, 21], but we do not know whether this effect also is seen on long-term outcomes. One may hypothesize, that an intervention with structured personal care may succeed to reduce the difference between socio-demographic groups [13], as the effectiveness of this intervention in two post hoc analyses have been reported to be especially pronounced in women [22] and in patients with severe mental illness [23].

Our aim in the present study is to describe how socio-demographic status and residence influences the effectiveness of an intervention with structured personal care in newly diagnosed patients with T2DM regarding all-cause mortality and any diabetes-related endpoint during 19 years of follow-up.

## Methods

### Patients

**Fig. 1:**
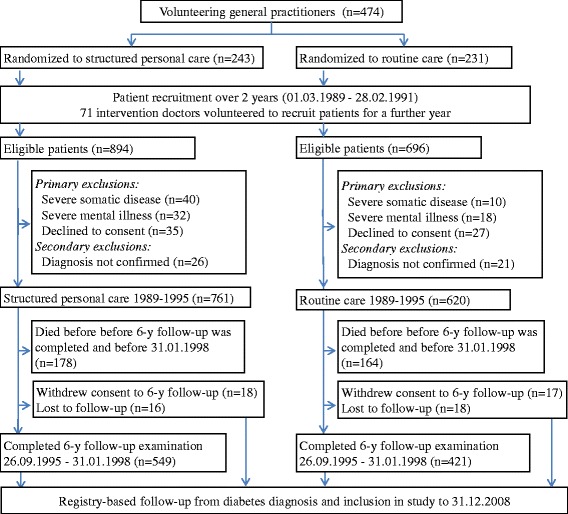
Patient flow diagram from inclusion until 19-year register based follow-up

The Diabetes Care in General Practice (DCGP) study was a pragmatic, open, multicentre, cluster-randomized controlled trial (ClinicalTrials.gov registration no. NCT01074762) [18]. The purpose of the trial was to test whether structured personal care, compared to routine care, for patients newly diagnosed with T2DM, reduced the incidence of seven pre-defined outcomes, including all-cause mortality and any diabetes-related outcome [18, 24]. In 1988, a random sample of two thirds of Danish general practices, excluding singlehanded practices with a doctor aged ≥60 years, received an invitation to participate in the study. Of 1902 general practitioners (GPs), 474 (25.4%) volunteered. In 1989–1992, all practices included all patients aged 40 years or over with newly diagnosed diabetes based on strict criteria [18]. All practices were randomized to either six years of structured personal care or routine care in the period 1989–1995 and all patients had a follow up examination 6 years after diagnosis [24]. The inclusion criteria were met by 1590 patients, of which 209 patients were excluded according to predefined criteria, so that the number of study participants was 1381 (Fig. 1). Among these patients, 1369 (99.1%) were of western European descent and, based on onset of insulin treatment, most were considered to have T2DM (97.5%). The Ethics Committee of Copenhagen and Frederiksberg (V.100.869/87) approved the study and all patients gave informed consent.

### Intervention

The Danish healthcare system is mainly tax-financed and based on the egalitarian principle of equal healthcare access for all equal healthcare needs, clinic visits are free of charge and expenditures for prescribed drugs are for the most part reimbursed. In Denmark the GP usually provides diagnosis and routine care for T2DM and function as gatekeeper for specialist care.

In the intervention arm follow-up every 3 months and annual screening for diabetes complications were supported by sending out a questionnaire to GPs one month before the next expected consultation. Goal-setting for blood glucose, blood pressure, lipids and weight was individualized [24], and GPs were introduced to possible solutions to therapeutic problems through six annual half-day seminars, descriptive annual reports on individual patients as well as folders and leaflets for doctors and patients, resembling present day recommendations for diabetes care [25, 26]. None of the intervention procedures were explicitly based on social differentials. Patients were never approached by the study centre. GPs in the routine care group were free to choose any treatment and change it over time [24] and they were not contacted after patient inclusion had stopped until the intervention was terminated and the 6-year examination was initiated in September 1995.

### Assessments

At the time of diagnosis and at the 6-year follow up examination measures of patients’ health and social status were collected in questionnaires to GPs, eye doctors and patients. Upon inclusion, patients gave information about socio-demographic variables in questionnaires: highest attained education level (basic school education only or higher education level); in labour market, out of labour market or retired; and whether patients lived alone or were cohabiting/married. Information on smoking habits and leisure-time physical activity was also collected. Information on place of living was recorded as rural or urban from area code and population density in accordance with Statistics Denmark [27].

### Clinical and registry based follow-up

The clinical 6-year follow-up examination included measurement of body weight, blood pressure, urinary albumin, haemoglobin A1c (HbA1c), total cholesterol, fasting triglycerides, and serum creatinine. Patients and doctors were asked to fill in questionnaires including questions on health behaviour (smoking, physical activity), attitudes towards disease (altered habits, diet and home monitoring of blood glucose), patient’s motivation for best possible control according to the GP, process-of-care (number of consultations, number of diabetes-related consultations and whether patients had been treated at a diabetes clinic) and if they received pharmacological treatment (cholesterol-lowering, glucose-lowering and antihypertensive drugs).

During 19 years (mean follow-up time) after diabetes diagnosis patients were followed up in the national registers. Vital and emigration status of all patients were certified through the Danish Civil Registration System [28]. Everyone living in Denmark is registered with a permanent and unique personal identification number allowing linkage between study populations and all national registers. Data on mortality, diagnoses and surgical procedures were from The Danish Register of Causes of Death (DCD) [29] and The Danish National Patient Register (DNPR), which includes information on almost all hospital contacts in Denmark [30]. The outcomes used in the registry-based follow up were all-cause mortality and any diabetes-related endpoint.

(e.g. stroke, myocardial infarction and renal failure, full list see (Additional file 1)), previously defined [18] and also used in the UK Prospective Diabetes Study [31].

### Statistical analysis

Differences in the incidence of death and any diabetes-related endpoint between randomization and socio-demographic groups were analysed univariably with log-rank tests and multivariably in Cox regressions models. In the latter, 95% CIs and *p* values were determined using a sandwich estimator for the variance to account for clustering of patients within practices [30]. Absolute risks were calculated as the number of participants experiencing the corresponding outcome divided by person years of risk. Two multivariable models are presented, one adjusted for age at diagnosis, sex and clustering, the other with additional adjustment for the following variables at diagnosis: BMI, hypertension, HbA1c, total cholesterol, urinary albumin, physical activity, smoking, known cardiovascular disease (see Table 1), and prescription of glucose- and/or lipid-lowering and/or antihypertensive drugs.

**Table 1:** Patient characteristics at diabetes diagnosis according to treatment arm

	No. of respondents Routine/ Structured care	Routine care	Structured care	*p*-value
Socioeconomic				
Age, years	620/761	65.2 (73.4; 56.2)	65.5 (73.7; 55.6)	0.59
Male gender	620/761	329 (53.1)	404 (53.1)	0.99
Living alone	606/743	198 (32.7)	236 (37.6)	0.72
Rural residence	596/728	161 (27.0)	153 (21.0)	0.011
Basic school education only	588/723	459 (78.1)	574 (79.4)	0.56
Employment status	605/744			0.51
In labor market		157 (26.0)	208 (28.0)	
Out of labor market^a^		181 (29.9)	203 (27.3)	
Retired		267 (44.1)	333 (44.8)	
Medical history				
Cardiovascular disease^b^	603/743	191 (31.7)	223 (30.0)	0.51
Clinical				
Body mass index, kg/m^2^	619/753	28.7 (32.2; 26.0)	29.4 (33.0; 26.2)	0.21
Hypertension^c^	620/761	458 (73.9)	568 (74.6)	0.75
Biochemical				
Fasting plasma glucose, mmol/L	620/761	13.8 (10.7; 17.1)	13.6 (10.7; 16.9)	0.44
HbA_1c_, %^d^	512/624	10.2 (8.7; 11.9)	10.2 (8.6; 11.7)	0.75
Total cholesterol, mmol/L	620/740	6.2 (5.5; 7.2)	6.2 (5.3; 7.1)	0.16
Fasting triglycerides, mmol/L	610/736	2.0 (1.4; 3.0)	2.0 (1.4; 2.9)	0.62
Serum creatinine, μmol/L	611/740	88 (79; 100)	90 (80; 101)	0.41
Urinary micro- or proteinuria^e^	595/723	254 (42.7)	304 (42.1)	0.81
Behavioral				
Sedentary (leisure-time) physical activity	604/741	162 (26.8)	210 (28.3)	0.54
Current smokers	604/742	208 (34.4)	264 (35.6)	0.66

Values are medians (interquartile range) or numbers (% of randomization group). The *p*-values are from Chi square tests for categorical data and Kruskal-Wallis tests for numeric data. ^a^Welfare benefits (unemployment-, social- health related benefits); ^b^Known cardiovascular disease: History of myocardial infarction, angina pectoris, stroke, intermittent claudication or amputation; ^c^Patients with systolic/diastolic BP > 160/90 mmHg and/or the use of antihypertensive and/or diuretic drugs; ^d^HbA_1c_ measured within 45 days of diabetes diagnosis, reference range 5.4–7.4% (59-81 mmol/mol); ^e^Protein level in urin > = 15 mg/L

For behavioural, clinical, biochemical and process-of-care variables at the 6-year examination we used multivariable generalized linear regression models (ordinary linear regression for continuous variables, logistic regression for binary variables and negative binomial regression for count variables) and presented the effects of structured care vs. routine care, stratified on sociodemographic groups adjusted for age, sex, and diabetes duration. Effect modification was assessed by a test for the interaction between randomization and SES groups in the corresponding model. Clustering with GPs was accounted for by the use of generalized estimating equations. Due to the multiple comparisons the level of statistical significance at 5% is not interpreted rigorously. We performed a log rank test for all-cause mortality and any diabetes-related endpoint comparing the four groups defined by educational background and the intervention. We used the statistical program SAS v9.4 (SAS Institute, Cary, NC, USA).

## Results

**Fig. 2:**
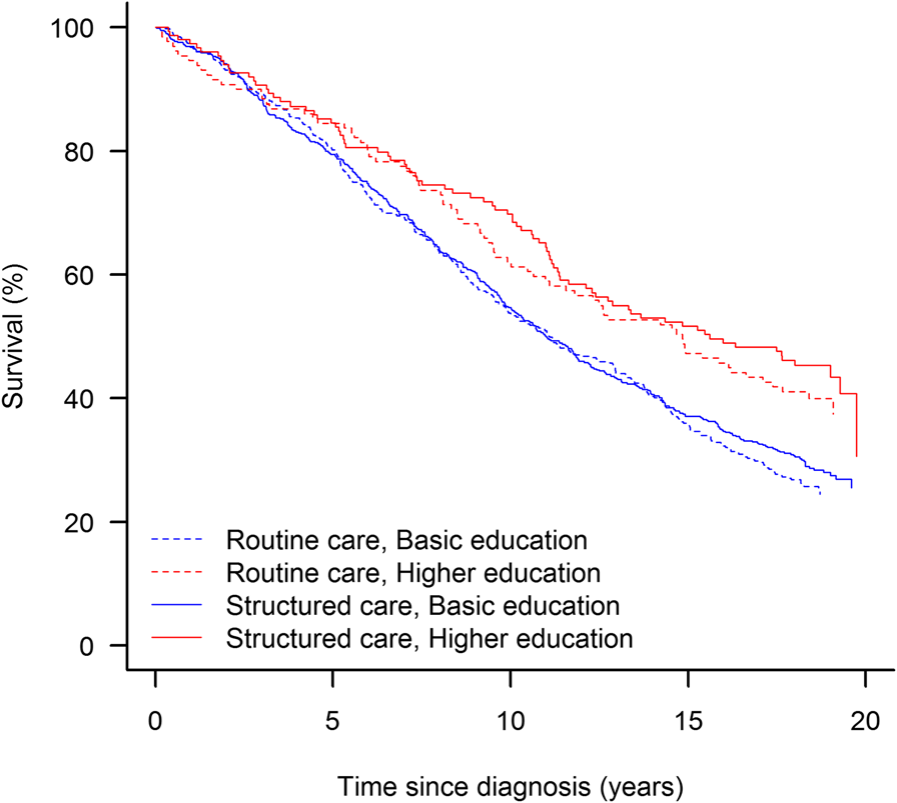
Kaplan-Meier plot showing the proportion of surviving patients since diabetes diagnosis in patients according to randomization arm and highest attained education level, only basic schooling vs. higher

**Fig. 3:**
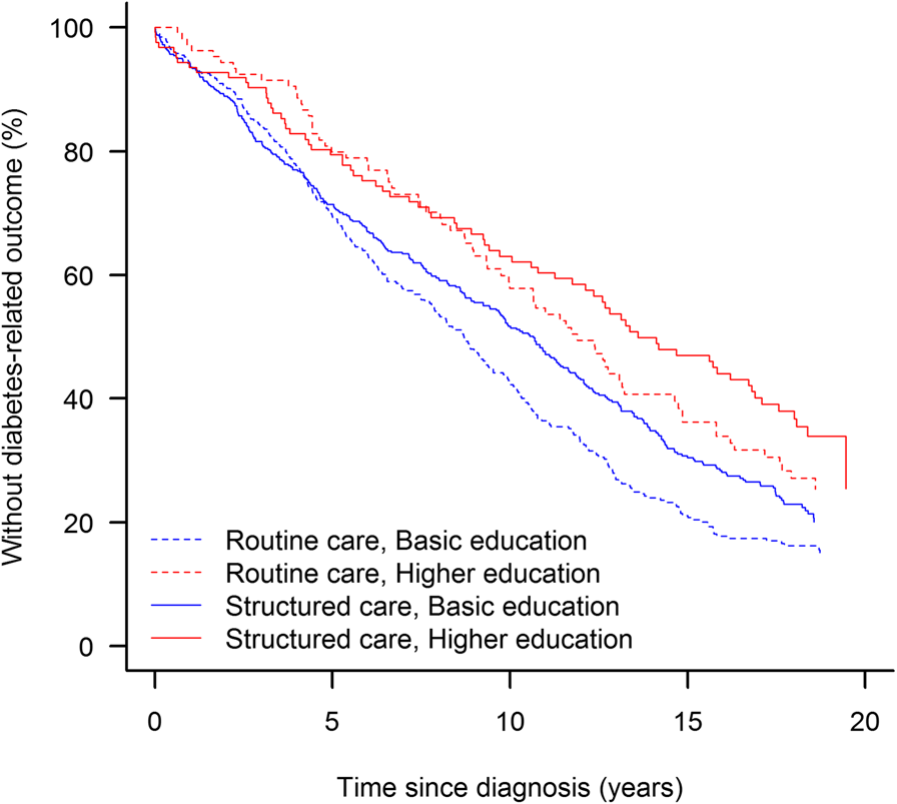
Kaplan-Meier plot showing the proportion of patients without any diabetes related endpoint since diagnosis according to randomization arm and highest attained education level, only basic schooling vs. higher

The indirect randomization was successful except that relatively more routine care patients were living in rural areas (Table 1). Overall, a low level of education was associated with higher all-cause mortality (Fig. 2) and any diabetes-related endpoint (Fig. 3) showing significant difference between the 4 groups in respectively all cause-mortality and any diabetes related endpoint (log-rank test, *p* < 0.0001). During 19 years of registry-based follow up structured personal care lowered the risk of any diabetes-related endpoint by 20% compared with routine care [18], and this effect seems to be most pronounced for patients living in urban areas (HR(95%CI) 0.71; 0.60–0.85), patients with only basic level of education (0.75; 0.63–0.89), patients outside the labour market (0.73; 0.55–0.97) and patients cohabiting (0.78; 0.65–0.93) (Table 2). The effect of the intervention, however, was only statistically significantly different between patients living in urban and rural areas (interaction *p* = 0.034). The intervention had no effect on all-cause mortality.

**Table 2:** Any diabetes-related outcome and all-cause mortality during 19 years of follow-up, according to socio-demographic group

	No. of patients without outcome at diabetes diagnosis (routine/ structured)	No. (%) of patients with outcome			Absolute risk (events per 1000 patient years)			Hazard ratio (HR)^a^ for structured- versus routine care (=reference group)				
		Routine care	Structured personal care	p value^b^	Routine care	Structured personal care	p value^c^	Adjusted for age, sex and clustering	p value^d^	Additionally adjusted for clinical and behavioral variables^f^	p value^d^	Interaction p value^e^
Education^f^ Any diabetes-related endpoint												0.11
Basic	377/461	281 (74.5)	313 (67.9)	0.04	89.2	74.5	0.01	0.80 (0.67–0.94)	0.008	0.75 (0.63–0.89)	0.0008	
Higher	108/124	72 (66.7)	74 (59.7)	0.27	61.5	53.0	0.31	0.94 (0.69–1.28)	0.69	1.01 (0.73–1.40)	0.97	
All-cause mortality												0.35
Basic	459/574	343 (74.7)	410 (71.4)	0.26	66.4	63.0	0.45	0.94 (0.81–1.08)	0.37	0.90 (0.77–1.06)	0.20	
Higher	129/149	78 (60.5)	84 (56.4)	0.56	47.8	43.0	0.48	1.03 (0.78–1.37)	0.84	1.06 (0.78–1.43)	0.73	
Employment status^g^ Any diabetes-related endpoint												0.50
Out of labor market	148/165	109 (73.7)	106 (64.2)	0.07	75.2	61.7	0.06	0.76 (0.58–1.01)	0.06	0.73 (0.55–0.97)	0.03	
In labor market	148/192	90 (60.8)	108 (56.3)	0.38	51.5	44.4	0.23	0.87 (0.67–1.14)	0.32	0.84 (0.63–1.12)	0.23	
All-cause mortality												0.87
Out of labor market	181/203	116 (64.1)	127 (62.6)	0.75	50.8	48.1	0.67	0.94 (0.74–1.20)	0.61	0.90 (0.69–1.18)	0.44	
In labor market	157/208	62 (39.5)	78 (37.5)	0.69	25.2	24.1	0.76	1.02 (0.74–1.39)	0.92	0.93 (0.66–1.31)	0.68	
Residence Any diabetes-related endpoint												0.033
Rural	137/133	94 (68.6)	89 (66.9)	0.09	67.1	77.7	0.24	1.01 (0.75–1.36)	0.95	1.07 (0.77–1.48)	0.70	
Urban	353/455	263 (74.5)	300 (65.9)	0.01	91.0	67.4	<0.0001	0.75 (0.63–0.88)	0.0007	0.71 (0.60–0.85)	0.0001	
All-cause mortality												0.21
Rural	161/153	108 (67.1)	112 (73.2)	0.28	54.0	64.9	0.14	1.09 (0.83–1.43)	0.55	1.08 (0.83–1.42)	0.56	
Urban	435/575	321 (73.8)	391 (68.0)	0.04	66.8	58.1	0.05	0.91 (0.78–1.06)	0.21	0.89 (0.75–1.05)	0.16	
Civil status Any diabetes-related endpoint												0.46
Living alone	158/176	120 (76.0)	127 (72.2)	0.42	101.6	92.7	0.41	0.83 (0.64–1.08)	0.16	0.88 (0.68–1.15)	0.36	
Cohabiting	344/426	244 (70.9)	273 (64.1)	0.04	74.4	62.4	0.02	0.83 (0.70–0.98)	0.03	0.78 (0.65–0.93)	0.007	
All-cause mortality												0.32
Living alone	198/236	167 (84.3)	195 (82.6)	0.62	87.5	85.1	0.80	0.84 (0.68–1.03)	0.09	0.84 (0.68–1.03)	0.09	
Cohabiting	408/507	267 (65.4)	314 (61.9)	0.31	52.6	49.3	0.41	1.00 (0.85–1.19)	0.98	0.97 (0.80–1.17)	0.71	

^a^The Hazard Ratio (HR), from a Cox proportional hazard regression model, reference group was routine care group. The corresponding 95% CIs and *p* values are determined using a sandwich estimator for the variance to account for clustering of patients within practices; ^b^ *p* value from a Rao-Scott χ^2^ test: a Pearson χ^2^ test adjusted for clustering of patients with general practitioners; ^c^ *p.* value from a log-rank test; ^d^Tests whether the effect of randomization differs between groups; ^e^ Adjusted for age at diagnosis, sex, clustering, BMI, hypertension, HbA_1c_, total cholesterol, urinary albumin, physical activity, smoking, known cardiovascular disease (see Table 1) and use of glucose- or lipid-lowering medication and antihypertensive therapy; ^f^ highest attained education level (Basic school education only/ higher level of education), ^g^ We excluded retired for this analysis

Results describing the role of four different aspects of socio-demography on behavioural, clinical, biochemical and process-of-care variables at the 6-year examination are presented in Additional file 2: Table S1, Additional file 3: Table S2, Additional file 4: Table S3, Additional file 5: Table S4, and summarized with the *p*-values from the test of the corresponding interaction and a description of the association (Table 3). Overall, the effect of the intervention on these variables did not differ according to socio-demography. Only few patients were treated with cholesterol lowering drugs in 1995–96 (1.9% - 5.8%), but relatively fewer patients living in rural areas were treated with cholesterol lowering drugs in response to the intervention compared to urban patients. The intervention resulted in an increased number of diabetes-related consultations regardless of socio-demographic group, and patients living alone tended to have relatively more consultations compared to patients cohabiting.

**Table 3:** The effect of structured personal care on behavioral, clinical, process of care and biochemical variables according to socio-demographic group

	Significance (p-value)^a^ of the modification of the intervention effect by the various socio-demographic variables			
	Educational level: Basic vs. higher	Residence: Rural vs. urban	Employment: Out of labor market vs. in labor market	Civil status: Single vs. cohabiting
Patient attitudes^b^				
Altered habits after diagnosis	0.22	0.08	0.50	0.33
Not full diabetes diet	0.53	0.74	0.78	0.45
Performs home blood/urinary glucose monitoring	0.17	0.89	0.94	0.88
For the patient in question the GP’s opinion^b^				
Patient’s motivation; good or very good for best possible control and treatment over past year^,^	0.34	0.07	0.19	0.80
Clinical				
Body mass index	0.29	0.50	0.15	0.65
Systolic blood pressure	0.47	0.23	0.71	0.54
Biochemical				
Hemoglobin A1c	0.48	0.31	0.91	0.97
Total cholesterol	0.05(↓)	0.67	0.83	0.79
Serum creatinine	0.42	0.05 (↓)	0.69	0.22
Micro- or proteinuria^c^	0.81	0.55	0.47	0.92
Behavioral^b^				
Sedentary (leisure time) physical activity	0.90	0.20	0.57	0.67
Current smoking	0.44	0.41	0.56	0.59
Process of care^b^				
Consultations/year	0.93	0.26	0.86	0.53
Diabetes-related consultations/year	0.91	0.18	0.56	0.04 (↑)
Ever treated at diabetic clinic	0.95	0.17	0.87	0.32
Glucose-lowering drugs^b^	0.26	0.96	0.60	0.33
Antihypertensive drugs^b^	0.33	0.18	0.55	0.98
Lipid-lowering drugs^b^	0.82	0.04 (↓)	0.40	0.40

^a^P-value from a test whether the effect of the intervention differs between socio-demographic groups (e.g. patients with basic school only vs higher education), adjusted for age, sex and diabetes duration. Clustering with general practice is accounted for by the use of generalized estimating equations. Arrows (↓) indicate the direction of the effect modification for cases where *p* < 0.05, e.g. the intervention lowered serum creatinine more in patients living in rural areas than in patients living in urban areas. ^b^Data from questionnaires to patients and their general practitioners. ^c^Proteinuria > =15 mg/L

## Discussion

The intervention did not influence all-cause mortality [18], but overall patients receiving structured personal care experienced a 20% lower risk of any diabetes-related endpoint compared to patients receiving routine care. This effect was greater among patients living in urban areas compared to rural patients, but otherwise, there was no effect modification of education, employment and civil status on the intervention for the final endpoints. Overall the effect of the intervention on behavioural, biochemical and process-of-care measures was not different between the subgroups of the four aspects of socio-demography.

### Comparison with existing literature

This study reports social inequity in mortality and diabetes-related morbidity, in line with other studies that have investigated the impact of level of education [9], occupation [8] and socio-economic status [7, 8]. The present findings suggest that an intervention with structured personal care does not give rise to more social inequity in use of the health care system, as has been described in other studies [13, 21]. Thus the results do not confirm results from studies of the general population showing that the number of consultations at the GP increases with decreasing socio-economic status [15, 32]. The fact that socio-economic differences in mortality and morbidity persist, despite formally equal access to the public health care system, could be due to different use of specialist care as suggested by others [17, 20], but this does not seem to be the case for the intervention in our study.

The presented benefit of structured personal care on long-term endpoints, also for patients with low level of education and patients on welfare benefits, cannot be ascribed to single elements of complex interventions. Patients with low level of education and income are frequently reported to be inadequately controlled with hyperglycaemia, hypertension, dyslipidaemia and unhealthy lifestyle [14, 15], and some studies also suggest socio-demographic differences in prescription rates [16, 20]. Our results are in line with earlier studies, concerning socio-demographic differences of intensive multifactorial interventions, which have been shown not to worsen or introduce social inequity in the control of T2DM [13, 21, 33]. Prior studies suggest that interventions seem to be effective, also among patient with low SES, in improving cardiovascular risk factors and prescription of medicine, but it is more difficult to assist patients in changing lifestyle and attitudes towards DM [13, 21, 33, 34]. As a high-risk group patients with low SES have potentially more to win [9, 10], but they are also known to have poor compliance especially regarding lifestyle and attitudes towards DM [5, 14, 15]. The individualization of diabetes care in the DCGP trial with negotiation of treatment goals, taking patient resources in consideration may explain why this intervention seems to be effective across the spectrum of SES.

Our results indicate that patients living in rural areas may have less benefit of the intervention compared to urban patients. An explanation for this finding could be because the uptake of the intervention was lower among rural doctors and/or because the compliance with the intervention among rural patients was low. We could not see any systematic differences in the 6 year follow up, on cardiovascular risk factors or behaviour between urban and rural patients that could reconcile these finding (Table 3). However, our data suggest that rural patients are less often treated with cholesterol-lowering drugs as a result of the intervention, but few patients were actually treated with this medication in the middle of the 1990s. Other studies have reported that cholesterol-lowering agents are less often prescribed in deprived areas [34]. A previous Scandinavian study reported no difference in mortality between urban and rural patients [8], like our study, other studies have suggested that people residing in more rural areas more often have undiagnosed diabetes [35, 36]. This implies that rural patients may be diagnosed at a later stage of the disease, possibly with a higher risk of complications and maybe less susceptible to treatment interventions than patients living in urban areas, this have to be investigated further.

### Strengths and weaknesses of the study

This study contributes to the knowledge on how SES and urbanisation influence the uptake and effect of diabetes interventions and may offer some advantages in comparison to prior studies. It is a strength of the present study that it reports hard endpoints after 19 years of follow up, but on the other hand a limitation that the results are from an early cohort.

Furthermore, the results are likely to be generalizable to the wider population of patients with T2DM because the study was population-based, with no upper age limit, but also because the study was conducted in general practice where most T2DM patients are treated. Also, the elements of the intervention, including the negotiation of treatment goals between patient and doctor, resemble standard treatment procedures in current day general practice diabetes care and recommendations [25, 26]. Finally, the study had a relative high number of GPs participating and the patient attrition rate was low.

The most important limitation of this study is that it is a post hoc analysis of a randomized controlled trial. Since randomization did not take socio-demography into account, this could have created imbalance between the randomization groups in relation to socio-demography, this does not seem to be the case. We although found a minor difference in randomization regarding residence that means one should be cautious when interpreting these results. The fact that we could not describe any substantial difference between rural and urban patients at the 6 years clinical follow up – means that the described potential difference in effect has to be investigated further. It is also a limitation that some elements of the intervention in the structured care arm were later applied to the routine care arm through national diabetes guidelines. The intervention could therefore prove to be more efficient than reported.

Information on educational level, employment and civil status were self-reported which might cause misclassification, but on the other hand, this information is readily at hand for the physician. Furthermore, some of the reported socio-demographic differences in mortality and morbidity could be due to patients with low level of education, outside the labour market or living alone being diagnosed later in the natural history of diabetes [9, 37] and therefore presenting with a more advanced disease at diagnosis [13]. Also a higher prevalence of comorbidity among patients with low SES in general could influence the result. We could however only find moderate decline in the association between low socioeconomic status and any diabetes-related endpoint when adjusting for comorbidity and baseline cardiovascular risk factors, which is in accordance with other studies [1, 11]. Long-term outcomes were registry-based and vital status was confirmed for all study participants. Not all non-fatal outcomes have been tested for validity, but this is generally considered to be acceptable [29, 30], and differential misclassification according to treatment allocation and socio-demography is not expected.

## Conclusion

Structured personal diabetes care showed effect on the aggregate outcome any diabetes-related endpoint, even for the high-risk group of patients with low level of education and patients out of the labour market. Thus, structured personal care did not give rise to *more* health inequality among patients with diabetes but rather a tendency in the opposite direction. Patients living in rural areas, however, seemed to have gained less from the intervention compared to patients living in urban areas this finding has to be investigated further. Still, socio-economic inequity in mortality and morbidity existed despite the intervention.

## Additional files

Additional file 1: Definition of clinical outcomes in the 19-year registry-based monitoring of the DCGP study, *any diabetes related endpoint* (list). (DOCX 76 kb)

Additional file 2: **Table S1.** The effect of structured personal care on behavioral, clinical, process of care and biochemical variables according to level of education (DOCX 35 kb)

Additional file 3: **Table S2.** The effect of structured personal care on behavioral, clinical, process of care and biochemical variables according to civil status (DOCX 37 kb)

Additional file 4: **Table S3.** The effect of structured personal care on behavioral, clinical, process of care and biochemical variables according to employment status (out of labor market vs. in labor market, retired excluded) (DOCX 37 kb)

Additional file 5: **Table S4.** The effect of structured personal care on behavioral, clinical, process of care and biochemical variables according to residence (Rural vs. Urban) (DOCX 37 kb)

## Abbreviations

BMI: Body Mass Index
CI: Confidence interval
DCD: Danish register of Causes of Death
DCGP: Diabetes in General Practice
DM: Diabetes mellitus
DNPR: Danish National Patient Register
GP: General Practitioner
HbA1c: Haemoglobin A1c
HR: Hazard Ratio
SES: Socio-economic status
T2DM: Type 2 Diabetes Mellitus
