# Supplementary material for: Socio-demographic determinants and effect of structured personal diabetes care: a 19-year follow-up of the randomized controlled study diabetes Care in General Practice (DCGP)
Source: BMC Endocr Disord. 2017 Dec 8;17:75. doi: 10.1186/s12902-017-0227-x (PMC5721594; doi:10.1186/s12902-017-0227-x)
Supplement: Supplementary file 1 — Definition of clinical outcomes in the 19-year registry-based monitoring of the DCGP study, any diabetes related endpoint (list). (DOCX 76 kb) [file 12902_2017_227_MOESM1_ESM.docx]

**Appendix 1**

Definition of clinical outcomes in the 19-year registry-based monitoring of the DCGP study, *any diabetes related endpoint*.

Sudden death

Death from hyperglycemia

Death from hypoglycemia

Fatal myocardial infarction

Nonfatal myocardial infarction

Angina/Ischemic heart disease

Heart failure

Fatal stroke

Nonfatal stroke

Nonfatal renal failure

Fatal renal disease

Amputation

Fatal peripheral vascular disease

Vitreous hemorrhage

Retinal photocoagulation

Blindness

Cataract excision

Codes used to classify cause of death or morbidity ICD 8 codes (≤1993) ICD-10 codes (≥1994) The Danish National Death Registry and the National Hospital Discharge Registry changed coding fromICD-8 to ICD-10 on January 1, 1994. The Danish National Death Registry contains only the first four characters of the ICD codes, while the National Hospital Discharge Registry contains all five characters.

Codes used to classify surgical procedures: The National Hospital Discharge Registry changed coding of surgical procedures from the third edition of The Danish Classification of Surgical Procedures to the Nordic Classification of Surgical Procedures on January 1, 1996.
