# Supplementary material for: Socio-demographic determinants and effect of structured personal diabetes care: a 19-year follow-up of the randomized controlled study diabetes Care in General Practice (DCGP)
Source: BMC Endocr Disord. 2017 Dec 8;17:75. doi: 10.1186/s12902-017-0227-x (PMC5721594; doi:10.1186/s12902-017-0227-x)
Supplement: Supplementary file 5 — Table S4. The effect of structured personal care on behavioral, clinical, process of care and biochemical variables according to residence (Rural vs. Urban) (DOCX 37 kb) [file 12902_2017_227_MOESM5_ESM.docx]

**Supplementary Table 4.** The effect of structured personal care on behavioral, clinical, process of care and biochemical variables according to residence (Rural vs. Urban)

|  | **Routine care (ref.)**  **/structured care** | | **Structured care**  **OR or mean diff**  (95% CI) | **p-value^**^** | **Routine care (ref.)**  **/structured care** | | **Structured care**  **OR or mean diff**  (95% CI) | **p-value^**^** | **Interaction P value^***^** |
| --- | --- | --- | --- | --- | --- | --- | --- | --- | --- |
| **Patient attitudes**^*^ |  | |  |  |  | |  |  |  |
| Altered habits after diagnosis, yes N (%), (OR) | 62 (59.1)/64 (67.4) | | 1.49 (0.90 ; 2.49) | 0.12 | 174 (63.7)/230 (60.7) | | 0.86 (0.62 ; 1.20) | 0.38 | 0.08 |
| Not full diabetes diet^,^ N (%), (OR) | 36 (34.0)/34 (35.4) | | 1.19 (0.66 ; 2.14) | 0.56 | 83 (30.3)/124 (32.8) | | 1.06 (0.76 ; 1.48) | 0.73 | 0.74 |
| Performs home blood/urinary glucose monitoring^,^ N (%), (OR) | 42 (39.6)/34 (35.4) | | 0.95 (0.50 ; 1.80) | 0.87 | 69 (25.7)/100 (26.4) | | 1.00 (0.67 ; 1.51) | 1.00 | 0.89 |
| **For the patient in question the GP’s opinion^*^** |  | |  |  |  | |  |  |  |
| Patient’s motivation; good or very good for best possible c control and treatment over past year^¤^ N (%), (OR) | 76 (69.1)/60 (57.7) | | 0.61 (0.37 ; 1.01) | 0.05 | 163 (56.2)/242 (58.2) | | 1.08 (0.76 ; 1.54) | 0.66 | 0.07 |
| **Clinical** |  | |  |  |  | |  |  |  |
| Body mass index, mean (SD), mean difference (kg/m2) | 28.92 (4.79)/28.26 (4.28) | | -0.38 (-1.68 ; 0.89) | 0.55 | 28.80 (5.09)/29.06 (5.12) | | 0.12 (-0.65 ; 0.89) | 0.76 | 0.50 |
| Systolic blood pressure, mean (SD), mean difference (mmHg) | 151.3 (19.7)/143.1 (22.6) | | -8.36 (-13.9 ; -2.85) | 0.003 | 152.1 (22.2)/147.9 (19.6) | | -4.38 (-7.7 ; -1.1) | 0.01 | 0.23 |
| **Biochemical** |  | |  |  |  | |  |  |  |
| Hemoglobin A1c, mean (SD), mean difference (%) | 9.3 (1.7)/8.9 (1.7) | | -0.3 (-0.7 ; 0.1) | 0.18 | 9.2 (1.7)/8.6 (1.5) | | -0.5 (-0.8 ; - 0.3) | <.0001 | 0.31 |
| Total cholesterol, mean (SD), mean difference (mmol/l) | 6.23 (1.16)/6.09 (1.20) | | -0.08 (-0.38 ; 0.21) | 0.58 | 6.15 (1.22)/6.02 (1.46) | | -0.16 (-0.36 ; 0.04) | 0.11 | 0.67 |
| Serum creatinine, mean (SD), mean difference (μmol/l) | 95.9 (27.2)/91.5 (15.2) | | -6.09 (-12.21 ; 0.03) | 0.05 | 96.8 (31.2)/98.8 (57.5) | | 3.10 (-3.51 ; 9.70) | 0.36 | 0.05 |
| Micro- or proteinuria >=15mg/L, N (%), (OR) | 44 (41.5)/35 (35.7) | | 0.74 (0.44 ; 1.24) | 0.25 | 113 (42.0)/150 (38.6) | | 0.89 (0.64 ; 1.24) | 0.49 | 0.55 |
| **Behavioral^*^** |  | |  |  |  | |  |  |  |
| *Sedentary* (leisure time) physical activity (OR) | 24 (22.9)/27 (28.1) | | 1.21 (0.63 ; 2.32) | 0.56 | 94 (34.3)/106 (28.1) | | 0.73 (0.51 ; 1.07) | 0.11 | 0.20 |
| Current smoking N (%), (OR) | 34 (32.4)/38 (40.0) | | 1.46 (0.81 ; 2.64) | 0.21 | 77 (28.3)/114 (29.8) | | 1.10 (0.78 ; 1.54) | 0.58 | 0.41 |
| **Process of care^*^** |  | |  |  |  | |  |  |  |
| Consultations/year, mean (SD), factor difference | 7.4 (6.0)/9.0 (10.5) | | 1.33 (1.01 ; 1.75) | 0.04 | 7.3 (5.4)/7.9 (5.1) | | 1.13 (1.02 ;1.26) | 0.02 | 0.26 |
| Diabetes-related consultations/year, mean (SD), factor difference | 4.2 (3.5)/5.4 (3.5) | | 1.40 (1.12 ; 1.74) | 0.003 | 4.4 (3.5)/5.0 (3.5) | | 1.19 (1.04 ; 1.39) | 0.01 | 0.18 |
| Ever treated at diabetic clinic, N (%), (OR) | 48 (42.9)/22 (21.2) | | 0.38 (0.20 ; 0.75) | 0.005 | 59 (20.3)/65 (15.6) | | 0.68 (0.43 ; 1.06) | 0.09 | 0.17 |
| **Glucose-lowering therapy** (oral/insulin), yes N (%), (OR) | 77 (68.8)/73 (70.2) | | 1.09 (0.59 ; 2.04) | 0.78 | 198 (68.3)/295 (70.7) | | 1.11 (0.79 ; 1.57) | 0.55 | 0.96 |
| **Antihypertensive therapy** N (%), (OR) | 62 (55.4)/51 (49.0) | | 0.74 (0.43 ; 1.29) | 0.29 | 152 (52.4)/233 (55.9) | | 1.16 (0.84 ; 1.59) | 0.37 | 0.18 |
| **Lipid lowering drugs** N (%), (OR) | 7 (6.3)/2 (1.9) | | 0.33 (0.06 ; 1.75) | 0.19 | 7 (2.4)/24 (5.8) | | 2.39 (0.94 ; 6.09) | 0.07 | 0.04 |
|  | |  | | | |  |  |  |  |

**Rural residence (N=216) Urban residence (N=924)**

* Data from questionnaires to patients or their general practitioners

**P-value from a t-test on the effect of structured care vs. routine care in an appropriate multivariate generalized linear model (ordinary linear regression for continuous variables, logistic regression for binary variables and negative binomial regression for count variables) adjusted for age, sex and diabetes duration. Clustering within general practitioners is accounted for by the use of generalized estimating equations.

***P-value from a t-test on the interaction effect between intervention and education level, i.e. a test whether the intervention effect differs between patients living in rural areas and patients living in urban areas, adjusted for age, sex and diabetes duration. Clustering with general practice is accounted for by the use of generalized estimating equations.
